# Occurrence and Human Health Risk Assessment of Pharmaceuticals and Hormones in Drinking Water Sources in the Metropolitan Area of Turin in Italy

**DOI:** 10.3390/toxics9040088

**Published:** 2021-04-20

**Authors:** Dimitra Papagiannaki, Stefania Morgillo, Gianluca Bocina, Paola Calza, Rita Binetti

**Affiliations:** 1 Società Metropolitana Acque Torino S.p.A.—Research Center, C.so Unità d’Italia 235/3, 10127 Turin, Italy; stefania.morgillo@smatorino.it (S.M.); gianluca.bocina@smatorino.it (G.B.); rita.binetti@smatorino.it (R.B.); 2 Department of Chemistry, University of Turin, Via Pietro Giuria 5, 10125 Turin, Italy; paola.calza@unito.it

**Keywords:** pharmaceuticals and hormones, occurrence, groundwater, raw drinking water sources, human health risk assessment

## Abstract

Pharmaceuticals and hormones (PhACs) enter the aquatic environment in multiple ways, posing potential adverse effects on non-target organisms. They have been widely detected in drinking water sources, challenging water companies to reassure good quality drinking water. The aim of this study was to evaluate the concentration of sixteen PhACs in both raw and treated drinking water sources in the Metropolitan Area of Turin—where Società Metropolitana Acque Torino (SMAT) is the company in charge of the water cycle management—and evaluate the potential human health risks associated to these compounds. Multivariate spatial statistical analysis techniques were used in order to characterize the areas at higher risk of pollution, taking into account the already existing SMAT sampling points’ network. Health risks were assessed considering average detected concentrations and provisional guideline values for individual compounds as well as their combined mixture. As reported in the just-issued Drinking Water Directive 2020/2184/UE, in order to establish priority substances, a risk assessment of contaminants present in raw drinking water sources is required for monitoring, identifying potential health risks and, if necessary, managing their removal. The results showed negligibly low human health risks in both raw water sources and treated water.

## 1. Introduction

The majority of European countries rely on surface and groundwater sources for their drinking water needs. However, the distribution of safe drinking water is one of the most important—although intricate—issues nowadays since these sources can often be contaminated. Surface and groundwater quality may be affected by both natural and anthropogenic factors [1]. Metals, single organic ions, more complex organic molecules, and biological components can derive from various sources, such as natural disasters, rural run-off, industrial and sewage discharge, population, and economic growth [1,2]. Water utilities and the scientific community are called to find efficient contaminants’ remediation systems in order to improve the performance of treatment plants and deliver safe drinking water to the population. The techniques usually in place include conventional methods such as precipitation, activated carbon adsorption, biological processes, and innovative methods such as advanced oxidation processes, membrane filtration using reverse osmosis, nano- and ultrafiltration processes, and biochar [3,4]. 

Pharmaceuticals and hormones (PhACs) represent one major category of anthropogenic contaminants present in the aquatic environment, degrading water quality [1]. In Europe, their use is continuously increasing, with 3000 compounds currently being active on the market [5]. Due to their large consumption, pharmaceuticals and hormones can reach the aquatic environment through different routes, including animal and human excretion, improper domestic or industrial discharge, and landfill leaching [1]. In the last decades, several studies reported their presence in surface, wastewater, and groundwater sources across Europe, in concentrations ranging from ng/L to a few μg/L [6,7,8,9]. In Italy, studies had mainly focused their attention on the detection of pharmaceuticals and hormones in surface and wastewater [7,10,11,12,13]. Hence, information about their presence in groundwater sources is still limited [14]. 

Wastewater treatment plants (WWTPs) represent an important remediation phase against contaminants’ release in the environment [15]. However, conventional treatment technologies used in WWTPs have been proven insufficient in removing PhACs from wastewater [16,17], setting them as one of the most important pollution sources. On the other hand, drinking water treatment plants (DWTPs) have a significant role in preventing unintended human exposure to them. Different studies have investigated the removal efficiency of PhACs through conventional and advanced treatment technologies used in drinking water treatment lines [18,19,20,21,22]. Their results showed difficulties in removing them completely, especially when they are present in trace levels. They are suggesting the need for combined treatment techniques to reassure safe quality of drinking water. 

The presence of PhACs in the water cycle is of great concern since they are responsible for ecological effects like toxicity and bioaccumulation on organisms or antibiotic resistance [23,24]. Little is known, though, about the potential adverse effects on human health that their occurrence in drinking water can cause, after long-term exposure to low doses. Although currently, studies focus on performing human health risk assessments, they have to take into account limitations such as inadequate datasets [14] and knowledge gaps concerning the synergistic effects of contaminants mixtures [25]. Due to the lack of this information, for most PhACs, regulations and drinking water guideline values have not been reported in Europe yet, and only a few are routinely monitored in the aquatic environment. To face these issues, in 2019, the European Union (EU) published the Strategic Approach for Pharmaceuticals in the Environment [26], focusing the attention on the need to improve their environmental monitoring, including risk assessments, management of waste, and identification of knowledge gaps. 

The aim of our study was to evaluate the sources and contamination levels of selected pharmaceuticals and hormones—included in the EU watchlist—in water bodies used as sources for drinking water in the Metropolitan Area of Turin (Italy), where Società Metropolitana Acque Torino (SMAT) is the company in charge of the water cycle management, and assess the potential risks to human health after long-term exposure to low concentrations. A selection of the sampling points, from those already existing in the SMAT network and usually included in monitoring campaigns in the area, was carried out prior to the analysis, based on their correlation with potential pollution sources, like hospitals and WWTPs. Finally, a human health risk assessment of each compound, as well as the mix of them, was done calculating provisional guideline values for those for which a drinking water guideline value did not exist. 

To the best of our knowledge, this is the first large-scale screening study in the Piemonte area in Italy reporting the occurrence of PhACs in raw surface, groundwater, and treated water, including a human health risk assessment of individual compounds and their mix.

## 2. Materials and Methods

### 2.1. Selection of Compounds

A priority list containing different pharmaceutical compounds and hormones was prepared based on the EU watch list, the just-issued European Drinking Water Directive (2020/2184/UE) [27], the Regional Environmental Protection Agency (ARPA Piemonte) analytical protocol and the NORMAN prioritization framework of emerging substances (Table A1). In this way, our conclusions led to sixteen different compounds, including antibiotics, beta-blockers, non-steroidal anti-inflammatory drugs, and hormones: Ketoprofen, Atenolol, Trimethoprim, Ofloxacin, Azithromycin, Ciprofloxacin, Cyclophosphamide, Sulfamethoxazole, Erythromycin, Clarithromycin, Diclofenac, Carbamazepine, Ibuprofen, Caffeine, Estrone, and 17-beta estradiol. Caffeine was included in this study as a tracer of anthropogenic pollution. 

### 2.2. Reagents and Chemicals

Stock solutions of the target compounds (Atenolol CAS 29122-68-7, Azithromycin CAS 83905-01-5, Caffeine CAS 58-05-2, Erythromycin CAS 114-07-8, Diclofenac sodium salt CAS 15307-79-6, Cyclophosphamide CAS 50-18-0, Ciprofloxacin CAS 85721-33-1, Sulfamethoxazole CAS 723-46-6, Carbamazepine CAS 298-46-4, Ketoprofen CAS 22071-15-4, Trimethoprim CAS 738-70-5, Clarithromycin CAS 81103-11-9, Ofloxacin CAS 82419-36-1, Estrone CAS 53-16-7, 17-beta Estradiol CAS 50-28-2, Ibuprofen CAS 15687-27-1; Sigma-Aldrich, St. Louis, MO, USA) were prepared in UHPLC-grade MeOH, purchased from Sigma-Aldrich, Co. (St. Louis, MO, USA). MilliQ water was obtained from MilliPore (MA, USA), LiChropur Formic Acid 98–100% and LiChropur Ammonia solution 25% for LC-MS were purchased from Merck KGaA (Darmstadt, Germany), Hydrochloric Acid (HCl), and Ethylenediaminetetraacetic acid trisodium salt dihydrate (Na_4_EDTA) were obtained from Fluka Analytical (Sigma-Aldrich, MO, USA), while Ammonium acetate for LC-MS was purchased from Fisher Chemical Scientific (Geel, Belgium).

### 2.3. Study Area and Sampling

**Figure 1:**
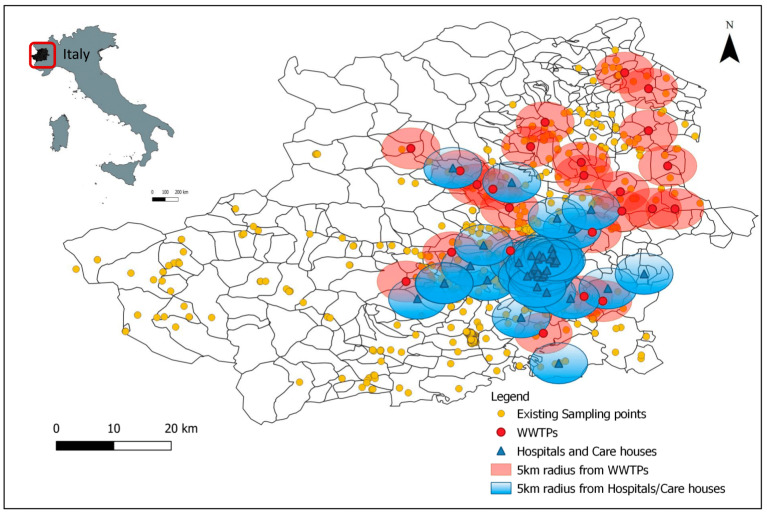
Map of the study area and its geographical position in the Italian territory, including all the SMAT existing sampling points in the catchment areas, WWTPs, hospitals/care houses taken into account as potential pollution sources.

The present study focused on the occurrence of pharmaceuticals and hormones in the Metropolitan Area of Turin (Piemonte, Italy), where SMAT is in charge of water distribution to 2.3 million inhabitants, supervising 293 municipalities. Within the context of Green Analytical Chemistry and for avoiding the costs, efforts and environmental impact of chemical analyses at a large-scale blind monitoring assessment, the selection of the sampling points based on the prioritization of the sites at major risk was done according to a geographical model, built in a previous study [28]. Spatial and multivariate statistical analysis tools were used in order to predict potential pollution levels and classify “hotspot” areas for monitoring. In this case, 44 hospitals and care houses and 24 major WWTPs in the territory were taken into account as possible pollution sources. As a result, 270 out of the 683 already existing sampling points in the catchment area (Figure 1)—used by SMAT for routine analyses—were found to be at highest risk and were selected for monitoring. 

The sampling/monitoring campaign lasted one year and was carried out between October 2019 and October 2020. In total, 328 samples were collected, according to the specifications and requirements of ISO 5667 accreditation [29], including groundwater, surface and drinking water. As surface water were considered the samples taken at the drinking water treatment plant’s intake, as drinking those taken after the last step of the whole treatment line, fountains, and tanks, and as groundwater those taken from pumps at each wellhead. Amber glass bottles (1 L)—previously decontaminated and rinsed with MeOH, according to the EPA 1694 method [30]—were used for water collection. The samples were refrigerated throughout their transport (10–15 °C), stored at 4 °C prior to their analysis, and analyzed within seven days from their sampling. 

### 2.4. Sample Preparation and Analysis 

For the extraction of the analytes (mainly acidic), the pH of water samples was adjusted to 2.0 with HCl after the addition of 500 mg of Na_4_EDTA to each of them. The 1 L samples were loaded to Oasis-HLB (200 mg) solid-phase extraction (SPE) cartridges (Waters, Milford, MA, USA)—which were preconditioned with 12 mL MeOH followed by 6 mL MilliQ and 6 mL MilliQ with pH 2.0—with a flow rate of 10 mL/min. The analytes were extracted from the sorbent material with 12 mL MeOH and reconstituted to 1 mL MilliQ after evaporating the solvent with a rotary evaporator (BUCHI Rotavapor R-114). Chromatographic analyses were carried out using a triple quadrupole SCIEX QTRAP^®^ 6500 system (SCIEX, Framingham, MA, USA) connected to a Thermo Scientific Dionex UltiMate 3000 HPLC system equipped with a Kinetex^®^ C18 HPLC column (1.7 μm particle size, 100 mm × 2.1 mm; Phenomenex Inc., Torrance, CA, USA). The QTRAP system operated in both Positive and Negative Electrospray Ionization Mode (ESI) using Multiple Reaction Monitoring (MRM) scan mode. Considering the heterogeneity among the compounds, three subsequent methods were developed. For the Positive ESI substances (Table A2), a volume of 12 µL of the sample was injected at a mobile phase consisted of a mixture of 0.1% Formic Acid in MilliQ Water and Methanol, following a gradient profile in a total run time of 10 min. For the Negative ESI compounds (Table A2), a volume of 10 µL of sample was injected at a mobile phase consisted of a mixture of 0.02% Ammonia in MilliQ Water and Methanol, following a gradient profile with a total run time of 10 min, while for Ibuprofen a volume of 10 μL was injected into a mixture of 0.1% Formic Acid and 0.1% Ammonium Acetate in MilliQ Water and Methanol, following a gradient profile in a total run time of 10 min (Table A2). 

### 2.5. Calculations 

#### 2.5.1. Validation Study

For reassuring the developed method’s applicability, a validation study was necessary and carried out according to ISO/IEC 17025 accreditation requirements [31]. Six-point calibration curves of final concentrations 1000, 2000, 4000, 6000 and 10,000 ng/L were built for each target compound and used for quantification taking into account the SPE preconcentration factor of 1000. For each point, fifteen replicates were analyzed and used for testing uncertainty, trueness, linearity, recovery and limits of Detection (LOD) and Quantification (LOQ). In addition, blank and quality control samples were analyzed to ensure the instrument’s best performance during the analysis. The quality control samples had a final concentration of 4000 ng/L, and their analysis was processed after every ten samples. The quantitation was performed using the MultiQuant^TM^ 3.0.3 software (SCIEX, Framingham, MA, USA).

#### 2.5.2. Average Concentrations in Water

In order to avoid the wrong estimation of the average detected concentrations of the compounds, non-detects were considered at a value of ¼ of the individual LOD of each target molecule as proposed by Houtman et al. [9]. This method was adopted since removing samples with non-detected compounds or setting their value as zero would have over or underestimated the average concentrations. 

#### 2.5.3. Human Health Risk Assessment 

As this study focused on the determination of selected pharmaceuticals and hormones in surface and groundwater for drinking water production, a human health risk assessment was necessary and was done by comparing the pharmaceuticals’ detected concentrations to guideline values. As a first step, we obtained the n-octanol-water partition coefficient (log Kow) for each compound using the KOWWIIN algorithm of the EPI Suite 4.11 software [32]. Compounds with log Kow > 3 were not included in the risk assessment study as there is a slighter possibility for them to pass through all the steps of the drinking water treatment line [33]. The Risk Quotient (RQ_i_) (Equation (1)) for each compound was then calculated as the ratio between the Mean Detected Concentration (MEC_i_) and the corresponding guideline value or, where it did not exist, the provisional guideline value ((p)GLV) [33]. The pGLVs were calculated using Equation (2),

RQ_i_ = MEC_i_/pGLV_i_, (1)

pGLV_i_ (μg/L) = [ADI × BW × 10% drinking water allocation]/DWI (2)

where ADI is the Acceptable Daily Intake (μg/kg bw/day); BW is the body weight set at a default value of 70 kg, as it is the closest to the average European bodyweight value of 70.8 kg [34]; DWI is the drinking water intake (L/day) set at a default value of 2 L/day as reported from WHO 2006 and a 10% of drinking water allocation factor was taken into account, as drinking water is not the only exposure way for humans [9,33,35]. The ADI values for the detected compounds were obtained from literature, and when they did not exist, they were derived from N(L)OAEL values by dividing them with an uncertainty factor of 100 [36]. RQ values ≥ 1 indicate the possibility of risk if the compound is ingested by drinking water consumption considering a lifelong exposure, while for RQ values ≤ 0.2 the risk for adverse human health effects is negligibly low [9,33]. Since in the majority of the samples more than two compounds occurred, a mixed health Risk Quotient (RQ_mix_) was calculated as a sum of individual RQs taking into account the Concentration Addition (CA) concept, as proposed by Qin et al. [37].

## 3. Results

### 3.1. Validation Results

Six-point calibration curves of a final range of 1000–10,000 ng/L—taking into account the preconcentration factor—were built and used for quantification and for defining linearity, trueness, uncertainty, recovery, LOD and LOQ for each target compound (Table 1). 

Good coefficient results were obtained for all the molecules (range 0.9951–0.9999), indicating a good linear correlation. Concerning the systematic and random errors, for uncertainty, accepted values were RSD ≤ 20%, for recovery within a range of 70–120% and for trueness ≤ 30%, following the ISO/IEC 17025 requirements [31]. Satisfying results within the required ranges were obtained for each point of the calibration curve, and those obtained for 4000 ng/L are reported in Table 1 (as an example). The recovery of the compounds after the off-line SPE treatment was checked in 4 different real water and 2 Milli-Q water samples spiked with the mix of the target compounds at two different concentrations (4 ng/L and 10 ng/L) and resulted in a range of 85.5–128% for all the compounds. Regarding the limits of Detection and Quantification, the guidelines of the ICH (International Conference on Harmonisation) Method [38] were followed. For calculating the LOD the ratio between the standard deviation of the y-intercepts of 15 replicates of the six-point calibration curve (taking into account the preconcentration factor of 1000) and the slope of the calibration curve was multiplied by 3.3, while the LOQ by multiplying 10 times the same ratio (Table 1). 

### 3.2. Screening Assessment in the Study Area

**Figure 2:**
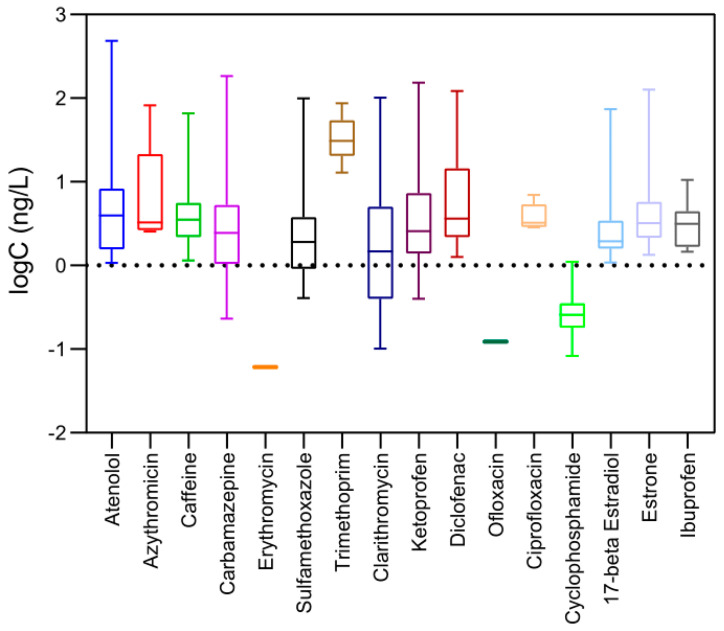
Boxplots showing the differences among the detected concentrations of the sixteen target PhACs in drinking water sources of the Metropolitan Area of Turin (Italy).

Within the context of Green Analytical Chemistry and in order to avoid a blind monitoring, a correlation study between the already existing sampling points in the area and the potential pollution sources was done based on a geographical model developed in another study [28]. Of note, 270 sampling points among the Metropolitan Area of Turin, including both surface and groundwater, resulted at a higher risk based on spatial regression, which correlated their geographical position with WWTPs, hospitals and care houses within a radius of 5 km, taking into account the nearest-neighbor points as well. In total, 325 samples were analyzed, 287 were groundwater and 24 were surface water. For raw samples—including both surface and groundwater matrices—in which the highest PhACs’ concentrations were detected, treated or finished water samples from the same areas were analyzed as well. In this way, 14 treated water samples were analyzed as well in order to reassure their good quality, and take the appropriate measurements if necessary. The average concentration detected in the area as a sum of the sixteen target compounds was 28.32 ng/L (ranging from 2.02 to 523.36 ng/L) in groundwater and 18.54 ng/L (2.02–82.05 ng/L) in surface water. In 40 samples, none of the target compounds was detected above their individual LOQs. Only one compound was detected in 52 samples, indicating that a mix of them was present in the majority of the samples. The maximum number of coexisting compounds was 11, and was detected only in one sample of groundwater. This sampling point is close to two WWTPs and one care house, indicating and confirming the high risk of pollution in this area again. The range of the individual detected concentrations in the study area was between 0.08 ng/L and 483.94 ng/L. Table 2 and Figure 2 summarize the occurrence concentrations for all the target compounds. 

From the sixteen target compounds included in this study, only two of them, ofloxacin and erythromycin, were not detected in any of the samples in concentrations higher than their individual LOQs (1.64 ng/L for ofloxacin and 0.81 ng/L erythromycin). The lack of detection results for these two compounds could depend on the human consumption trends in the area or on the compounds’ physicochemical characteristics, enabling them to be adsorbed or biodegraded. These results are in accordance with a study from Verlicchi et al. [10], that did not detect ofloxacin and erythromycin above their individual method detection limits (MDL) in surface water from the Po Valley (Italy). On the other hand, the most abundant compounds were caffeine and ketoprofen. Caffeine was detected in 176 groundwater samples with an average concentration of 4.61 ng/L (1.15–65.92 ng/L), and in 23 surface water samples at an average concentration of 5.34 ng/L (1.31–61.28 ng/L). Caffeine is generally reported as one of the most abundant compounds in the aquatic environment worldwide. However, the concentrations found in this study are significantly lower than those reported in other studies (in the scale of μg/L) [39]. Moreover, ketoprofen was detected in 143 groundwater samples with an average concentration of 6.51 ng/L (0.16–152.98 ng/L), and in 21 surface water samples at an average concentration of 5.84 ng/L (0.43–71.84 ng/L). The wide range of the detected concentrations for ketoprofen is also confirmed from other studies in highly urbanized areas in Italy [12,39] and could be correlated to socioeconomic aspects. 

Concerning hormones, estrone was detected in 117 groundwater samples with an average concentration of 4.03 ng/L (1.09–125.97 ng/L), and in 12 surface water samples at an average concentration of 1.49 ng/L (1.30–8.33 ng/L). The other target hormone, 17-beta estradiol, was detected in 114 raw surface and groundwater samples with an average concentration of 1.28 ng/L. 17-beta estradiol is the only compound from those included in the study that is subjected to a guideline (1 ng/L). In 24 of the analyzed samples, only a mix of the two hormones 17-beta estradiol and estrone was present, while none of the two was detected in 148 of them. 

The highest detected concentrations in this study were for atenolol 483.94 ng/L, estrone 125.97 ng/L, carbamazepine 183.49 ng/L, ketoprofen 152.88 ng/L, and diclofenac 121.46 ng/L. All of them were detected in groundwater samples around WWTPs, highlighting the need to implement new removal techniques. In general, the findings of this study were in accordance with the literature reporting occurrence patterns in Italy [11,12,13,39] and in other countries as well [9,15,18,40,41,42,43]. However, in order to better estimate the impact of PhACs in the studied area, further information on their occurrence through time is needed. Additional monitoring campaigns are already planned in order to better assess the risks these molecules can cause. 

### 3.3. Occurrence of Pharmaceuticals in Treated/Drinking Water

Even if a limited number of studies examining the occurrence of PhACs in drinking water are available, their existence has been confirmed in tap water around the globe [19,20,21,40]. Moreover, these studies claimed that conventional treatments in DWTPs like flocculation and sedimentation are unable to remove PhACs from water completely, especially when present in trace levels (in the order of ng/L) [18]. An improvement of treatment lines including steps, like ozonation and adsorption with activated carbon, is necessary in order to remove them. Hence, the best solution for drinking water companies in order to reassure safe and good quality of drinking water is a combination of them in their treatment lines [15,44]. SMAT as a drinking water company has incorporated this approach in particular for more vulnerable water resources. In such cases, DWTPs include multiple steps such as pre-settling, ozonation, clarification-flocculation, oxidation, filtration with activated carbon and/or ultrafiltration and final disinfection. 

**Figure 3:**
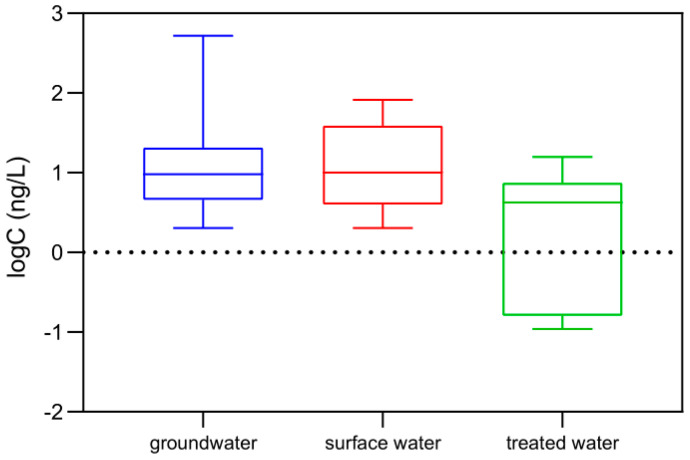
Boxplots showing differences among the sums of detected concentrations in different water type samples, including groundwater (*n* = 287), surface (*n* = 24) and treated water (*n* = 14).

Treated samples originating from both surface and groundwater have been included in this study as well and the results showed that atenolol, azithromycin, erythromycin, sulfamethoxazole, trimethoprim, diclofenac, ofloxacin, ciprofloxacin, cyclophosphamide, clarithromycin, estrone, and 17-beta-estradiol were not quantified at all, even if most of them were present in the raw water. The reasons for their absence could be due to different phenomena: biodegradation, adsorption on the carbon filters and oxidation, mainly chlorination [15,18]. On the other hand, even if carbamazepine, caffeine, ibuprofen and ketoprofen have been detected in some treated samples in concentrations above their individual LOQs, they were still at a very low level. One parameter—other than the consumption trends in the area—that could explain their existence in treated water, is their hydrophilic behavior (log Kow < 3.0), since those with higher log Kow values are expected to be adsorbed on the particles and removed through the treatment line steps. These results have been confirmed by other studies as well, which report the occurrence of these compounds after the treatment lines [15,36]. Figure 3 highlights the differences between the sum of detected concentrations in raw and treated water samples and the good degree of efficiency of the applied treatments. 

### 3.4. Human Health Risk Assessment

#### 3.4.1. Individual Compounds

Taking into account the calculation of the log Kow values, only ten out of the sixteen target compounds of this study were considered as potential threats to human health if present in drinking water. This assumption was confirmed by analyzing samples after treatment with ozonation, GAC filtration and chlorination, which resulted in negligible low or zero concentrations of PhACs. However, ketoprofen and ibuprofen—even if with log Kow values higher than 3—were included in the risk assessment as well since they were detected in treated samples.

pGLV values could not be derived from toxicological data in the literature—confirming knowledge gaps in PhACs risk assessment estimation [45]—and they were calculated using ADI values and, where not existing, N(L)OAEL values. All the ADI and N(L)OAEL values were obtained from literature and the most restrictive value was used (Table 3). The pGLVs ranged from 0.07 μg/L for ofloxacin to 5285 μg/L for the psychoactive compound caffeine. The RQ_average_ for every compound was calculated as the ratio between the derived pGLV value and the mean detected concentration in raw water sources, while the RQ_max_ as the ration between the pGLV value and the maximum detected concentration for each compound. All determined RQis were lower than 0.2 (Table 3), indicating that none of the target compounds could potentially pose a risk of adverse health effects to humans even after a lifelong exposure. Even if most of the target compounds were present in surface and groundwater samples in the area, their quantification frequency was low, indicating low probability of threat to human health. These outcomes are in accordance with other studies, which report that the majority of the detected contaminants in drinking water sources do not pose individually a risk to human health [6,9,33,35,46]. 

Among the target compounds, the two hormones, estrone and 17-beta estradiol, are well known for their endocrine disruptive activity. Their presence in surface and groundwater sources could result in severe risks to human health. To prevent these negative health effects from their possible occurrence in drinking water, chlorination and ozonation have been reported as efficient remediation technologies [47]. In this study, 17-beta estradiol and estrone have not been detected in the treated water samples for drinking water consumption, as in the studied DWTP both treatment techniques occur, confirming the literature’s results. Hence, human health risks from the two hormones are not reported in the study. 

**Table 3:** Human health risk assessment parameters for target PhACs in raw water matrices.

Compounds	Log Kow	ADI (μg/kg bw/day)	Source	pGLV (μg/L)	MEC (ng/L)	RQi_average_	RQi_max_
Atenolol	−0.03	2	[48]	7	2.29	3.27 × 10^−4^	6.91 × 10^−2^
Azithromycin	3.24	Not considered	N/A	N/A	Not considered	N/A	N/A
Caffeine	0.16	1510	[49]	5285	3.52	6.65 × 10^−7^	1.25 × 10^−5^
Carbamazepine	2.25	0.34	[48]	1.19	2.63	2.21 × 10^−3^	1.54 × 10^−1^
Clarithromycin	3.18	Not considered	N/A	N/A	Not considered	N/A	N/A
Ciprofloxacin	−0.001	12	[48]	42	0.37	8.75 × 10^−6^	1.67 × 10^−4^
Cyclophosphamide	0.97	33	[48]	115.5	0.11	9.80 × 10^−7^	9.52 × 10^−6^
Diclofenac	0.57	200	N/A	700	1.5	2.14 × 10^−6^	1.74 × 10^−4^
Erythromycin	2.48	0.7	[50]	2.45	0.06	0	0
Ketoprofen	3.00	20	[36]	70	4.07	5.81 × 10^−5^	2.18 × 10^−3^
Ofloxacin	−0.20	0.02	[48]	0.07	0.12	0	0
Sulfamethoxazole	0.48	510	[48]	1785	1.39	7.78 × 10^−7^	5.57 × 10^−5^
Trimethoprim	0.73	190	[48]	665	1.78	2.68 × 10^−6^	1.31 × 10^−4^
17-beta Estradiol	3.94	Not considered	N/A	N/A	Not considered	N/A	N/A
Estrone	3.43	Not considered	N/A	N/A	Not considered	N/A	N/A
Ibuprofen	3.79	400	[36]	1400	0.2	1.52 × 10^−7^	7.53 × 10^−6^

#### 3.4.2. Risk Assessment of Combined Exposure

Since in the majority of the samples more than one PhAC was present, an estimation of the risk only for individual compounds could result in risk underestimation [36,51]. However, since toxicological data of mixtures are limited, in this study we calculated the RQ of the mixtures as a sum of the individual RQs of the detected compounds according to the concentration addition (CA) concept [51]. This concept is widely used for calculating the combined risks of exposure and it assumes that the different compounds in the mixture will not interact among them, since they share the same mechanism of action and the same toxicity target [25,37]. All the components in a mixture contribute to the total toxicity depending on their concentration, resulting to the expectation that even if the individual compounds do not pose a risk, the mixture could pose it due to the addition effect [25]. This assumption was confirmed by the results of this study, that showed a risk of the combined exposure higher than the individual, but negligibly low as well (lower than 0.2). Although only some PhACs were taken into account in this study, usually a larger number of pollutants exists in the aquatic environment—even in trace levels—highlighting the fact that the mixture risk assessment is incomplete and further research is needed in order to find new ways of estimating it [37,44].

## 4. Conclusions

This study has addressed, in the first place, the presence of pharmaceuticals and hormones in surface and ground water in the Metropolitan Area of Turin (Italy, Piemonte). Prior to the screening assessment, a correlation study was performed in order to identify the areas at higher contamination risk and the good quality of the criteria employed was confirmed by the results obtained. Fourteen out of the sixteen compounds analyzed have been detected at low concentration ranging from tens to hundreds of ng/L. Since these water resources are used as catchment areas for drinking water production, a human health risk assessment was included. The results showed that risk for adverse human health effects was negligibly low—both for individual compounds and the mixture of them—in water sources before treatment, and almost non-existent in treated/finished drinking water. Nevertheless, the results of this study can be relevant for the prioritization of hazardous substances (as reported in the just-issued Drinking Water Directive (2020/2184/UE) in order to address suitable monitoring campaigns and any necessary countermeasures to be adopted for safeguarding these essential resources. Finally, they could be used for filling the knowledge gaps and attract attention to the need for regulations aimed at reducing the spread of pharmaceuticals and hormones in the environment and, in particular, in natural water resources, which is a major concern worldwide.

## Figures and Tables

**Table 1:** Validation results for every target compound.

Compounds	Conc. (ng/L)	Trueness % n = 15	Uncertainty % n = 15	Linearity	LOD (ng/L)	LOQ (ng/L)
Atenolol	4000	−3.977	3.047	0.9996	0.196	0.655
Azithromycin	4000	−10.090	7.290	0.9951	0.736	2.454
Caffeine	4000	−1.300	1.912	0.9991	0.322	1.073
Carbamazepine	4000	−14.831	9.761	0.9999	0.066	0.219
Clarithromycin	4000	−3.519	2.786	0.9996	0.031	0.074
Ciprofloxacin	4000	−1.603	0.892	0.9996	0.788	2.625
Cyclophosphamide	4000	−0.161	2.563	0.9996	0.010	0.034
Diclofenac	4000	−7.627	2.531	0.9998	0.376	1.254
Erythromycin	4000	−4.793	3.114	0.9998	0.244	0.814
Ketoprofen	4000	−10.221	4.476	0.9999	0.115	0.385
Ofloxacin	4000	−1.1769	2.735	0.9978	0.493	1.644
Sulfamethoxazole	4000	−4.823	2.202	0.9983	0.110	0.366
Trimethoprim	4000	−7.457	5.497	0.9998	3.492	11.369
17-beta Estradiol	4000	−7.129	6.546	0.9972	0.303	1.010
Estrone	4000	−23.144	3.655	0.9971	0.400	1.333
Ibuprofen	4000	−1.599	1.770	0.9969	0.412	1.375

**Table 2:** Occurrence concentrations of target PhACs in the study area, without taking into account the non-detected.

Compounds	QF * n = 325	C_min_ (ng/L)	C_max_ (ng/L)	C_average_ (ng/L)	C_median_ (ng/L)	Q1 (ng/L)	Q3 (ng/L)
Atenolol	12.00%	1.07	483.94	18.73	3.96	1.64	7.93
Azithromycin	4.00%	2.55	82.46	14.84	3.28	2.64	14.63
Caffeine	61.23%	1.15	65.92	5.69	3.53	2.21	5.51
Carbamazepine	37.84%	0.23	183.49	6.93	2.44	1.07	5.24
Clarithromycin	22.46%	0.10	101.30	7.57	1.48	0.40	4.60
Ciprofloxacin	4.30%	2.86	7.00	4.16	3.25	2.88	5.33
Cyclophosphamide	9.23%	0.08	1.10	0.31	0.26	0.19	0.34
Diclofenac	11.38%	1.26	121.46	12.41	3.62	2.22	11.89
Erythromycin	0.00%	<LOD	<LOQ	<LOD	<LOD	<LOD	<LOD
Ketoprofen	48.92%	0.4	152.88	8.28	2.58	1.40	7.31
Ofloxacin	0.00%	<LOD	<LOQ	<LOD	<LOD	<LOD	<LOD
Sulfamethoxazole	27.69%	0.41	99.47	4.94	1.91	0.92	3.68
Trimethoprim	2.46%	12.87	87.16	37.80	31.02	22.07	41.71
17-beta Estradiol	35.07%	1.08	9.00	1.28	1.18	1.45	2.04
Estrone	36.00%	1.35	125.97	7.69	3.20	2.12	5.71
Ibuprofen	3.07%	1.46	10.54	3.77	3.15	1.78	3.73

* QF = Quantification Frequency (calculated as the ratio between the number of samples with detected concentrations of PhACs and the total number of analyzed samples).

## Appendix A

**Table A1:** Analysis parameters for the 16 target compounds.

Compound	Chemical Group	Regulation Status	Drinking Water Value
Atenolol	β-Blockers	NORMAN framework prioritization ^1^	N/A
Azithromycin	Macrolide Antibiotic	EU Watch List	N/A
Clarithromycin		EU Watch List	N/A
Erythromycin		EU Watch List	N/A
Caffeine	Stimulant	NORMAN framework prioritization	N/A
Carbamazepine	Anticonvulsant	NORMAN framework prioritization	N/A
Ciprofloxacin	Fluoroquinolones antibiotics	NORMAN framework prioritization	N/A
Ofloxacin		NORMAN framework prioritization	N/A
Cyclophosphamide	Alkylating agent	NORMAN framework prioritization	N/A
Diclofenac	Analgesics anti-inflammatory drugs	EU Watch List	N/A
Ketoprofen		NORMAN framework prioritization	N/A
Ibuprofen		NORMAN framework prioritization	N/A
Sulfamethoxazole	Antibacterial sulfonamides	NORMAN framework prioritization	N/A
Trimethoprim		NORMAN framework prioritization	N/A
17-beta Estradiol	Estrogens	EU Watch List	1 ng/L (reference value)
Estrone		NORMAN framework prioritization	N/A

^1^https://www.norman-network.net/ (accessed on 04 September 2019).

**Table A2:** Analysis parameters for the 16 target compounds.

HPLC Method	Ionization Mode	Compounds
0.1% Formic Acid in MilliQ Water and Methanol, total run time: 10 min, flow rate: 0.200 mL/min	Positive ESI	Atenolol
		Azithromycin
		Caffeine
		Carbamazepine
		Clarithromycin
		Ciprofloxacin
		Cyclophosphamide
		Diclofenac
		Erythromycin
		Ketoprofen
		Ofloxacin
		Sulfamethoxazole
		Trimethoprim
0.02% Ammonia in MilliQ Water and Methanol, total run time: 10 min, flow rate: 0.400 mL/min	Negative ESI	17-beta Estradiol
		Estrone
0.1% Formic Acid and 0.1% Ammonium Acetate in MilliQ Water and Methanol, total run time: 10 min, flow rate: 0.400 mL/min	Negative ESI	Ibuprofen

## References

[1] Richardson S.D., Kimura S.Y. (2020). Water Analysis: Emerging Contaminants and Current Issues. Anal. Chem..

[2] Khatri N., Sanjiv T. (2015). Influences of Natural and Anthropogenic Factors on Surface and Groundwater Quality in Rural and Urban Areas. Front. Life Sci..

[3] Fuoco I., Figoli A., Criscuoli A., Brozzo G., De Rosa R., Gabriele B., Apollaro C. (2020). Geochemical Modeling of Chromium Release in Natural Waters and Treatment by RO/NF Membrane Processes. Chemosphere.

[4] Palansooriya K.N., Yang Y., Tsang Y.F., Sarkar B., Hou D., Cao X., Meers E., Rinklebe J., Kim K.-H., Ok Y.S. (2020). Occurrence of Contaminants in Drinking Water Sources and the Potential of Biochar for Water Quality Improvement: A Review. Crit. Rev. Environ. Sci. Technol..

[5] Patel M., Kumar R., Kishor K., Mlsna T., Pittman C.U., Mohan D. (2019). Pharmaceuticals of Emerging Concern in Aquatic Systems: Chemistry, Occurrence, Effects, and Removal Methods. Chem. Rev..

[6] Baken K.A., Sjerps R.M.A., Schriks M., van Wezel A.P. (2018). Toxicological risk assessment and prioritization of drinking water relevant contaminants of emerging concern. Environ. Int..

[7] Castiglioni S., Fanelli R., Calamari D., Bagnati R., and Zuccato E. (2004). Methodological approaches for studying pharmaceuticals in the environment by comparing predicted and measured concentrations in River Po, Italy. Regul. Toxicol. Pharmacol..

[8] Calza P., Medana C., Padovano E., Giancotti V., Minero C. (2013). Fate of selected pharmaceuticals in river waters. Environ. Sci. Pollut. Res..

[9] Houtman C.J., Kroesbergen J., Lekkerkerker-Teunissen K., van der Hoek J.P. (2014). Human health risk assessment of the mixture of pharmaceuticals in Dutch drinking water and its sources based on frequent monitoring data. Sci. Total Environ..

[10] Verlicchi P., Al Aukidy M., Jelic A., Petrović M., Barceló D. (2014). Comparison of measured and predicted concentrations of selected pharmaceuticals in wastewater and surface water: A case study of a catchment area in the Po Valley (Italy). Sci. Total Environ..

[11] Zuccato E., Castiglioni S., Fanelli R., Reitano G., Bagnati R., Chiabrando C., Pomati F., Rosseti C., Calamari D. (2006). Pharmaceuticals in the environment in Italy: Causes, occurrence, effects and control. Environ. Sci. Pollut. Res. Int..

[12] Patrolecco L., Capri S., Ademollo N. (2015). Occurrence of selected pharmaceuticals in the principal sewage treatment plants in Rome (Italy) and in the receiving surface waters. Environ. Sci. Pollut. Res. Int..

[13] Al Aukidy M., Verlicchi P., Jelic A., Petrovic M., Barcelò D. (2012). Monitoring release of pharmaceutical compounds: Occurrence and environmental risk assessment of two WWTP effluents and their receiving bodies in the Po Valley, Italy. Sci. Total Environ..

[14] Meffe R., de Bustamante I. (2014). Emerging organic contaminants in surface water and groundwater: A first overview of the situation in Italy. Sci. Total Environ..

[15] Santos A.V., Couto C.F., Lebron Y.A.R., Foureaux A.F.S., Reis E.O., Santos L.V., de Andrade L.H., Amaral M.C.S., Lange L.C. (2020). Occurrence and risk assessment of pharmaceutically active compounds in water supply systems in Brazil. Sci. Total Environ..

[16] Pedrouzo M., Borrull F., Pocurull E., Marcé R.M. (2011). Presence of Pharmaceuticals and Hormones in Waters from Sewage Treatment Plants. Water Air Soil Pollut..

[17] Pal P. (2018). Treatment and Disposal of Pharmaceutical Wastewater: Toward the Sustainable Strategy. Sep. Purif. Rev..

[18] Huerta-Fontela M., Galceran M.T., Ventura F. (2011). Occurrence and removal of pharmaceuticals and hormones through drinking water treatment. Water Res..

[19] Jiang X., Qu Y., Liu L., He Y., Li W., Huang J., Yang H., Yu G. (2019). PPCPs in a drinking water treatment plant in the Yangtze River Delta of China: Occurrence, removal and risk assessment. Front. Environ. Sci. Eng..

[20] Vulliet E., Cren-Olivé C., Grenier-Loustalot M.-F. (2011). Occurrence of pharmaceuticals and hormones in drinking water treated from surface waters. Environ. Chem. Lett..

[21] Simazaki D., Kubota R., Suzuki T., Akiba M., Nishimura T., Kunikane S. (2015). Occurrence of selected pharmaceuticals at drinking water purification plants in Japan and implications for human health. Water Res..

[22] Snyder S.A. (2007). Removal of EDCs and Pharmaceuticals in Drinking and Reuse Treatment Processes.

[23] Lagesson A., Fahlman J., Brodin T., Fick J., Jonsson M., Byström P., Klaminder J. (2016). Bioaccumulation of five pharmaceuticals at multiple trophic levels in an aquatic food web—Insights from a field experiment. Sci. Total Environ..

[24] Chen C., Hilaire S., Xia K., Waldrip H.M., Pagliari P.H., He Z. (2020). Veterinary Pharmaceuticals, Pathogens and Antibiotic Resistance. Animal Manure.

[25] Heys K.A., Shore R.F., Pereira M.G., Jones K.C., Martin F.L. (2016). Risk assessment of environmental mixture effects. RSC Adv..

[26] EUR-Lex, European Union Strategic Approach of Pharmaceuticals in the Environment. https://eur-lex.europa.eu/legal-content/EN/TXT/HTML/?uri=CELEX:52019DC0128&from=EN.

[27] EUR-Lex, DIRECTIVE (EU) 2020/2184. https://eur-lex.europa.eu/legal-content/EN/TXT/HTML/?uri=CELEX:32020L2184&from=EN.

[28] Binetti R., Calza P., Costantino G., Morgillo S., Papagiannaki D. (2019). Perfluoroalkyl Substance Assessment in Turin Metropolitan Area and Correlation with Potential Sources of Pollution According to the Water Safety Plan Risk Management Approach. Separations.

[29] ISO (2009). ISO 5667 Water Quality—Sampling.

[30] EPA Method 1694 (2007). Pharmaceuticals and Personal Care Products in Water, Soil, Sediment, and Biosolids by HPLC/MS/MS.

[31] ISO (2017). ISO/IEC 17025 General Requirements for the Competence of Testing and Calibration Laboratories.

[32] US EPA (2012). Estimation Programs Interface Suite™ for Microsoft® Windows.

[33] Schriks M., Heringa M.B., van der Kooi M.M.E., de Voogt P., van Wezel A.P. (2010). Toxicological relevance of emerging contaminants for drinking water quality. Water Res..

[34] Walpole S.C., Prieto-Merino D., Edwards P., Cleland J., Stevens G., Roberts I. (2012). The weight of nations: An estimation of adult human biomass. BMC Public Health.

[35] de Jongh C.M., Kooij P.J.F., de Voogt P., ter Laak T.L. (2012). Screening and human health risk assessment of pharmaceuticals and their transformation products in Dutch surface waters and drinking water. Sci. Total Environ..

[36] Pais M.C.N., Nascimento E.D.S. (2018). Guideline values and human risk assessment for the presence of anti-inflammatory drugs remaining in drinking water after lab scale treatment. Braz. J. Pharm. Sci..

[37] Qin L.-T., Pang X.-R., Zeng H.-H., Liang Y.-P., Mo L.-Y., Wang D.-Q., Dai J.-F. (2020). Ecological and human health risk of sulfonamides in surface water and groundwater of Huixian karst wetland in Guilin, China. Sci. Total Environ..

[38] Guideline, ICH Harmonised Tripartite (2005). Validation of Analytical Procedures: Text and Methodology Q2 (R1).

[39] Castiglioni S., Davoli E., Riva F., Palmiotto M., Camporini P., Manenti A., Zuccato E. (2018). Data on occurrence and fate of emerging contaminants in a urbanised area. Data Brief.

[40] Loos R., Locoro G., Comero S., Contini S., Schwesig D., Werres F., Balsaa P., Gans O., Weiss S., Blaha L. (2010). Pan-European survey on the occurrence of selected polar organic persistent pollutants in ground water. Water Res..

[41] Bexfield L.M., Toccalino P.L., Belitz K., Foreman W.T., Furlong E.T. (2019). Hormones and Pharmaceuticals in Groundwater Used As a Source of Drinking Water Across the United States. Environ. Sci. Technol..

[42] Ebele A.J., Oluseyi T., Drage D.S., Harrad S., Abou-Elwafa Abdallah M. (2020). Occurrence, seasonal variation and human exposure to pharmaceuticals and personal care products in surface water, groundwater and drinking water in Lagos State, Nigeria. Emerg. Contam..

[43] Lv J., Zhang L., Chen Y., Ye B., Han J., Jin N. (2019). Occurrence and distribution of pharmaceuticals in raw, finished, and drinking water from seven large river basins in China. J. Water Health.

[44] Yang Y., Ok Y.S., Kim K.-H., Kwon E.E., Tsang Y.F. (2017). Occurrences and removal of pharmaceuticals and personal care products (PPCPs) in drinking water and water/sewage treatment plants: A review. Sci. Total Environ..

[45] World Health Organization (2012). Pharmaceuticals in Drinking-Water.

[46] Ben Y., Fu C., Hu M., Liu L., Wong M.H., Zheng C. (2019). Human health risk assessment of antibiotic resistance associated with antibiotic residues in the environment: A review. Environ. Res..

[47] Teresa O.L.d.V.M., Jessica A.-L., Isaura Y.-N. (2020). Assessing the Estrogenic Activity of EDCs and Human Risks of Groundwater after Ozonation and Chlorination. Ozone Sci. Eng..

[48] Bruce G.M., Pleus R.C., Snyder S.A. (2010). Toxicological Relevance of Pharmaceuticals in Drinking Water. Environ. Sci. Technol..

[49] Caffeine—ECHA. https://echa.europa.eu/registration-dossier/-/registered-dossier/10085/7/6/2.

[50] Drugs@FDA: FDA-Approved Drugs. https://www.accessdata.fda.gov/scripts/cder/daf/.

[51] Backhaus T., Faust M. (2012). Predictive environmental risk assessment of chemical mixtures: A conceptual framework. Environ. Sci. Technol..

